# Cloning and Expression of the *γ*-Polyglutamic Acid Synthetase Gene* pgs*BCA in* Bacillus subtilis* WB600

**DOI:** 10.1155/2016/3073949

**Published:** 2016-03-17

**Authors:** Biaosheng Lin, Zhijuan Li, Huixia Zhang, Jiangwen Wu, Maochun Luo

**Affiliations:** ^1^College of Life Science, Longyan University, Longyan 364012, China; ^2^Fujian Provincial Key Laboratory of Preventive Veterinary Medicine and Veterinary Biotechnology, Longyan University, Longyan 364012, China

## Abstract

To clone and express the *γ*-polyglutamic acid (*γ*-PGA) synthetase gene* pgs*BCA in* Bacillus subtilis*, a pWB980 plasmid was used to construct and transfect the recombinant expression vector pWB980-*pgs*BCA into* Bacillus subtilis* WB600.* PgsBCA* was expressed under the action of a P43 promoter in the pWB980 plasmid. Our results showed that the recombinant bacteria had the capacity to synthesize *γ*-PGA. The expression product was secreted extracellularly into the fermentation broth, with a product yield of 1.74 g/L or higher. *γ*-PGA samples from the fermentation broth were purified and characterized. Hydrolysates of *γ*-PGA presented in single form, constituting simple glutamic acid only, which matched the characteristics of the infrared spectra of the *γ*-PGA standard, and presented as multimolecular aggregates with a molecular weight within the range of 500–600 kDa. Expressing the *γ*-PGA synthetase gene* pgsBCA* in* B. subtilis* system has potential industrial applications.

## 1. Introduction

Gamma-polyglutamic acid (*γ*-PGA) is a new water-soluble biodegradable material. It is an anionic polypeptide formed by the condensation of amide linkages between *α*-amino and *γ*-carboxylic acid groups of the D- and/or L-glutamate in microorganisms. It has nontoxic, edible, adhesive, film-forming, and moisture retention properties [1]. *γ*-PGA and its derivatives can be used as drug carriers and bioadhesive materials that have been widely used in pharmaceutical, cosmetics, food, agriculture, and sewage treatment industries and have become one of the most interesting topics in biopolymer research [2].

Traditionally, *γ*-PGA is primarily produced through microbial fermentation [3]. Bacteria involved in *γ*-PGA synthesis are mostly gram-positive (genus:* Bacillus*, class:* Bacilli*) and are classified as glutamate-dependent or glutamate nondependent types based on their needs for glutamate [4]. Wild-type *γ*-PGA-producing strains have unstable heritability, easily leading to a reduction or loss in the ability to synthesize *γ*-PGA during fermentation, undergo *γ*-PGA degradation, and produce extracellular polysaccharide by-products, thereby lowering product yield. Compared to traditional mutation breeding, genetic engineering technologies have been expected to become an effective method to create *γ*-PGA high-yield strains. Ashiuchi et al. [5] and Tarui et al. [6] confirmed that* pgs*B,* pgs*C, and* pgs*A are three essential genes involved in *γ*-PGA synthesis in glutamate-dependent strains. Urushibata et al. [7] and Jiang et al. [8] constructed recombinant plasmids containing the* pgs*BCA gene through different methods of fusion expression and further transformed the plasmids into* Escherichia coli* to obtain positive clones that were capable of producing *γ*-PGA.* E. coli*, a gram-negative bacterium, has been reported as the primary host strain for transforming the recombinant vector of the *γ*-PGA synthase gene. However, its synthase gene is mainly derived from* Bacillus subtilis* (gram-positive bacteria). The membrane structures and protein secretion systems of both types of bacteria vary, which in turn may result in poor positioning of the recombinant expressed *γ*-PGA synthase system on the bacterial cell membrane [9]. Therefore, the level of expression of *γ*-PGA in the host strain is lower; and the amount of *γ*-PGA obtained from positive clones is only within the range of 0.024–0.134 g/L [10].* B. subtilis*, as a prokaryotic expression host for food safety, carries some excellent features in expressing *γ*-PGA that* E. coli* does not possess. For example,* B. subtilis* is capable of expressing the soluble and nonfusion proteins, as well as preferentially expressing the nonpathogenic and nonapparent codons [11]. In addition, its expression of a recombinant plasmid after transformation is high. Therefore, its expression products have greater advantages and higher potential in manufacturing biological engineering products for the food and pharmaceutical industries. However, the relevant study of cloning and expression of* pgs*BCA in* B. subtilis* was comparatively scarce. To date, the expression of the *γ*-PGA synthase gene* pgs*BCA still need D-xylose and L-arabinose induced, generally with poor expression yield and low molecular weight (only 200–500 kDa) [12], indicating the need to resolve this particular bottleneck. Considering this, in this paper, the recombinant plasmid expressing* pgs*BCA gene was reconstructed and highly expressed in* B. subtilis* as to improve the yield and molecular weight of *γ*-PGA.* B. subtilis* 168 has been widely used in the study of *γ*-PGA regulation. It is one of the few bacterial strains that has a complete set of *γ*-PGA synthase genes but does not produce *γ*-PGA [13]. The present study used the genomic DNA of* B. subtilis* 168 as DNA template to amplify the *γ*-PGA synthase gene* pgs*BCA and to further clone the* pgs*BCA gene into the* B. subtilis* expression vector pWB980 to transform into type strain* B. subtilis* WB600. We constructed a recombinant* B. subtilis* expression system for *γ*-PGA synthesis, which may serve as a foundation for the high-yield industrial production of *γ*-PGA based on an engineered* B. subtilis* expression system.

## 2. Materials and Methods

### 2.1. Bacterial Strains and Plasmids

*B. subtilis* 168 and* B. subtilis* WB600 were purchased from Shanghai Genemy BioTech Co., Ltd. (Shanghai, China);* E. coli* JM109 was prepared and preserved at our laboratory and described in a previous study. pMD19-T vector and* B. subtilis* expression vector pWB980 were purchased from TakaRa Biotechnology (Dalian) Co., Ltd. (Dalian, China).

### 2.2. Reagents

All restriction endonucleases, T4 DNA ligase,* Taq* DNA polymerase, dNTPs, DNA ladder marker, and protein molecular weight markers, were purchased from TakaRa Biotechnology (Dalian) Co., Ltd. Plasmid extraction and agarose DNA extraction kits were purchased from Tiangen Biotech (Beijing) Co., Ltd. (Beijing, China). Bacterial genomic DNA extraction kits were purchased from and primers were designed and synthesized by Sangon Biotech (Shanghai) Co., Ltd. (Shanghai, China). Silica gel plates for thin layer chromatography (TLC) were purchased from Qingdao Jiyida Silica Reagent Factory (Model number: 50 × 100 GF254, Shandong, China).

### 2.3. Culture Medium

Lysogeny broth (LB) was prepared using 10 g/L tryptone, 5 g/L yeast extract, and 10 g/L NaCl (pH 7.0) and 2.0% (W/V) agar powder to solidify the medium.* E. coli* and* B. subtilis* transformants were selected with 50 *μ*g/mL ampicillin (Amp^r^) and 30 *μ*g/mL kanamycin (Km^r^), respectively. Fermentation broth for the genetically engineered recombinant bacteria contained 40 g/L glucose, 0–100 g/L sodium glutamate, 6 g/L (NH_4_)_2_SO_4_, 2 g/L K_2_HPO_4_, and 0.2 g/L MgSO_4_ (pH 7.5).

### 2.4. Primer Design

With reference to the NCBI database, the upstream and downstream* pgs*B,* pgs*C, and* pgs*A coding gene sequences of* B. subtilis* 168 were designed as follows: BAC1: 5′-CGCGGATCCATGTGGTTACTCATFATAGCC-3′ (restriction site of* Bam*HI endonuclease is underlined); BAC2: 5′-CCCA AGCTTTTATTTAGATTTTAGTTTGTCA C-3′ (restriction site of* Hind*III endonuclease is underlined).

### 2.5. Cloning of *γ*-PGA Synthetase Gene

*B. subtilis* 168 genomic DNA was used as template. BAC1 and BAC2 primers were used to amplify the gene. The PCR reaction system included 2 *μ*L of DNA template, 10 *μ*L of 5x buffer, 2 *μ*L of dNTPs, 2 *μ*L of individual primers of BAC1 and BAC2, 0.5 *μ*L of 5x Primer STAR, and sterile double-distilled water to prepare a final volume of 50 *μ*L. Reaction conditions were as follows: 94°C for 3 min, followed by 30 cycles of 94°C for 30 s, 55°C for 15 s, and 72°C for 3 min, and a final 72°C extension for 10 min. One percent agarose gel electrophoresis was used to identify the PCR reaction products. PCR products were recovered using a DNA rapid recovery reagent and ligated into the pMD19-T vector, which was followed by transformation into* E. coli* JM109 competent cells using CaCl_2_ methods. The selected single colonies were inoculated into liquid LB to expand the plasmid. Intermediate vectors pMD-*pgs*BCA were then obtained and identified using* Bam*HI and* Hind*III double digestion as well as sequencing.

### 2.6. Construction of* B. subtilis* Expression Vector

**Figure 1:**
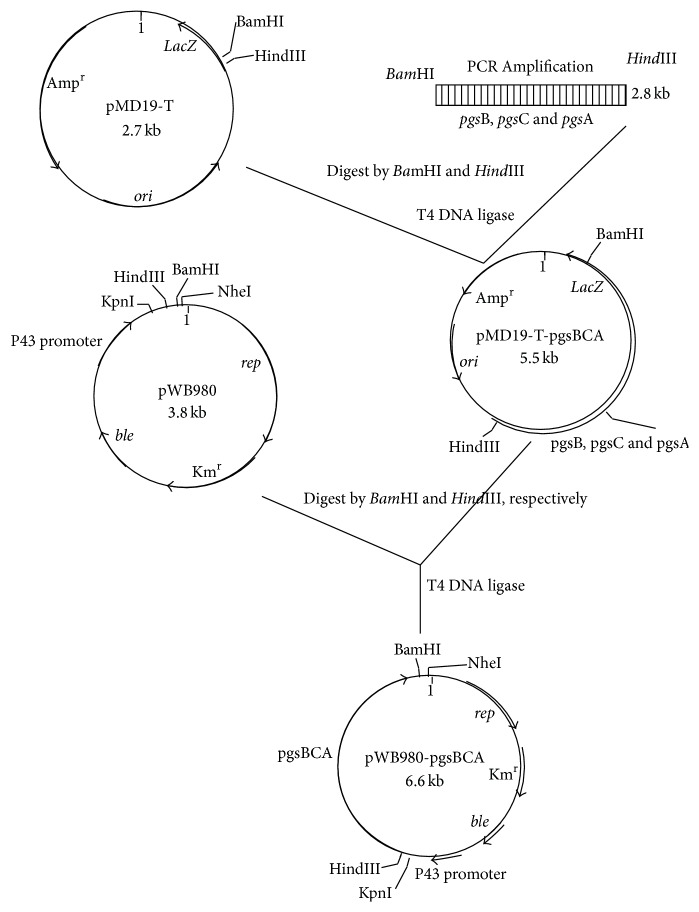
Construction of recombinant plasmid pWBb980-*pgs*BCA from* Bacillus subtilis* expression vector pWB600 and *γ*-*pgs*BCA gene.

*Bam*HI and* Hind*III double digestion was performed to cut the intermediate vector pMD-T-*pgs*BCA and pWB980 plasmid, followed by ligating these into the recombinant expression vector, pWB980-*pgs*BCA (Figure 1). Kanamycin resistance screening was performed to screen the recombinant plasmid, followed by plasmid extraction and identification using restriction enzyme digestion and sequencing to obtain the positive clones of the bacterial strain.

### 2.7. Induced Expression of* pgs*BCA Gene

pWB980-*pgs*BCA plasmids were transformed into* B. subtilis* WB600 to obtain recombinant strains of* Bacillus* WB600-*pgs*BCA, which were inoculated into 5 mL of fresh liquid LB containing 30 *μ*g/mL kanamycin and incubated at 37°C in a 200 rpm shaker overnight. The next day, a 2% inoculum of the culture suspension was further inoculated into 250 mL flask with 100 mL recombinant fermentation medium containing kanamycin and incubated at 37°C in a 200 rpm shaker for 36–48 h until the bacterial concentration stopped growing and fermentation was terminated. pWB980-*pgs*BCA contained a constitutive P43 promoter. Hence, we did not add any inducers during the fermentation process. Approximately 0–100 g/L sodium glutamate was added into the fermentation medium as a synthetic substrate for *γ*-PGA to further study the impact of different substrate concentrations on the synthetic yield of *γ*-PGA.

### 2.8. *γ*-PGA Isolation and Purification

After adding the optimal substrate concentration and fermentation had ended, the fermentation medium was centrifuged at 5,000 rpm for 5 min to collect the supernatant. The supernatant was mixed with 4 volumes of absolute ethanol and left to stand overnight at 4°C, followed by centrifugation at 4,000 rpm, and then the supernatant was discarded. The pellet was redissolved in the appropriate amount of distilled water and further centrifuged at 5,000 rpm to obtain the supernatant. A 20 mg/mL solution of proteinase K was added into the supernatant and dialyzed overnight using deionized water. After the centrifugation as earlier described, the supernatant was collected and freeze-dried to obtain the purified solid samples of *γ*-PGA. *γ*-PGA samples were stored at −70°C until analysis.

### 2.9. Hydrolysis of *γ*-PGA

A 0.5 g purified *γ*-PGA sample was added to 10 mL of 6 moL/L HCl, vacuumed for 10 min, and then sealed. The sample was then hydrolyzed at 110°C for 12–24 h, allowed to cool down and then filtered, and redissolved in 6 moL/L of NaOH to adjust the pH to 7.0. The aqueous solution was transferred to a 100 mL flask, and the hydrolysate was subjected to TLC using silica gel plates to analyze its amino acid composition.

### 2.10. Determination of *γ*-PGA Contents and Properties

*γ*-PGA contents of fermentation broth were measured by high-performance liquid chromatography (HPLC) [14]. The purified *γ*-PGA samples underwent infrared spectroscopy using Shimadzu's IR Prestige-21 infrared spectrometer, Shimadzu (China) Co., Ltd. (Beijing, China). Potassium bromide (KBr) was used as reference material [15]. The molecular weight of *γ*-PGA was determined by SDS-PAGE [16].

## 3. Results

### 3.1. PCR Amplification and Identification of *γ*-PGA Synthetase Gene* pgs*BCA

**Figure 2:**
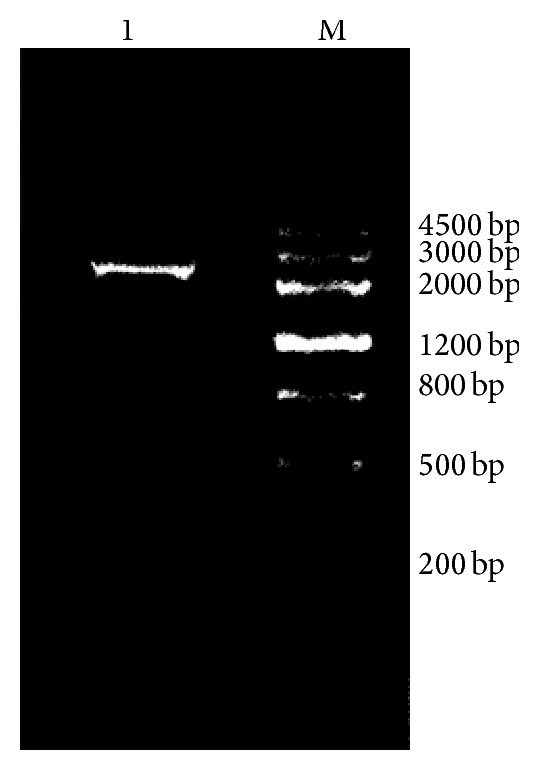
PCR product of* pgs*BCA gene. Note: Lane 1,* pgs*BCA PCR product; Lane M, DNA markerIII (Tiangen).

The target gene was amplified by PCR.  Figure 2 shows the PCR products that were separated and analyzed using agarose gel electrophoresis. The observed size of the amplified* pgs*BCA fragment, 2.8 kb, was in agreement with our expected results. An agarose DNA extraction kit was used to recover and purify the PCR products. After confirming with DNA sequencing, the DNA sequence of the PCR products was determined to be 100% identical with the sequence of the reported gene of* B. subtilis* 168.

### 3.2. Identification of* B. subtilis* Expression Vectors

**Figure 3:**
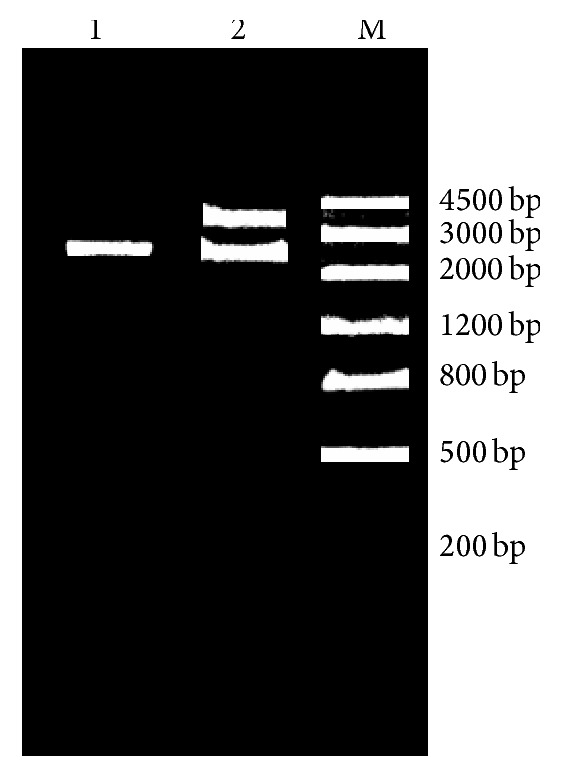
Map of electrophoresis of recombinant plasmid pWB980-*pgs*BCA after digestion. Note: Lane 1,* pgs*BCA PCR product; Lane 2, after double digestion of pWB980-*pgs*BCA with* Bam*HI and* Hind*III; Lane M, DNA markerIII (Tiangen).

After transforming the constructed recombinant expression vectors, pWB980-*pgs*BCA, into competent cells, the plasmids were collected and identified using* Bam*HI and* Hind*III restriction enzyme digestions.  Figure 3 shows that, as shown in the map of double restriction enzyme digestions, the size of the cleaved fragment was the same as that of the* pgs*BCA PCR products, thereby initially confirming the successful construction of the recombinant expression vector, pWB980-*pgs*BCA.

### 3.3. Impact of Different Substrate Concentrations on the Synthetic Yield of *γ*-PGA

**Figure 4:**
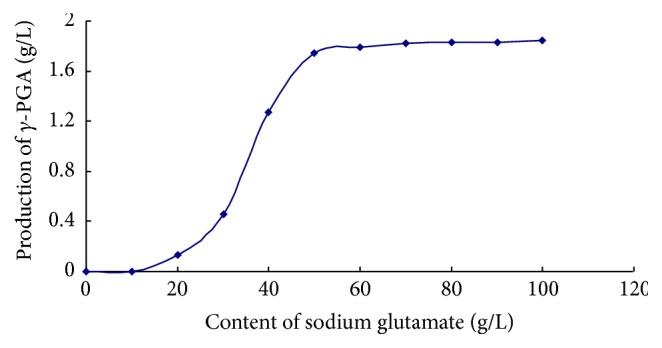
Production of *γ*-PGA in fermentation of recombinants (g/L). As increasing amounts of the substrate, glutamate, the production of *γ*-PGA was enhanced. However, when glutamate concentration was >50 g/L, the synthetic yield of *γ*-PGA declined.

Figure 4 shows that, with increasing amounts of the substrate, glutamate, the production of *γ*-PGA was enhanced. However, when glutamate concentration was >50 g/L, the synthetic yield of *γ*-PGA declined. This result suggested that* pgs*BCA was secreted by* B. subtilis* WB600. The expressed product, *γ*-PGA, could be secreted into extracellular fermentation broth. Using a lower substrate concentration, we observed that the recombinant bacteria did not synthesize *γ*-PGA, indicating that an excess amount of substrate was necessary for the recombinant bacteria to synthesize *γ*-PGA. Therefore, from the perspective of economic efficiency, we identified that a substrate concentration of 50 g/L was optimal to synthesize the highest possible amount of *γ*-PGA (1.74 g/L).

### 3.4. Characterization of Recombinant *γ*-PGA in Fermentation Broth

**Figure 5:**
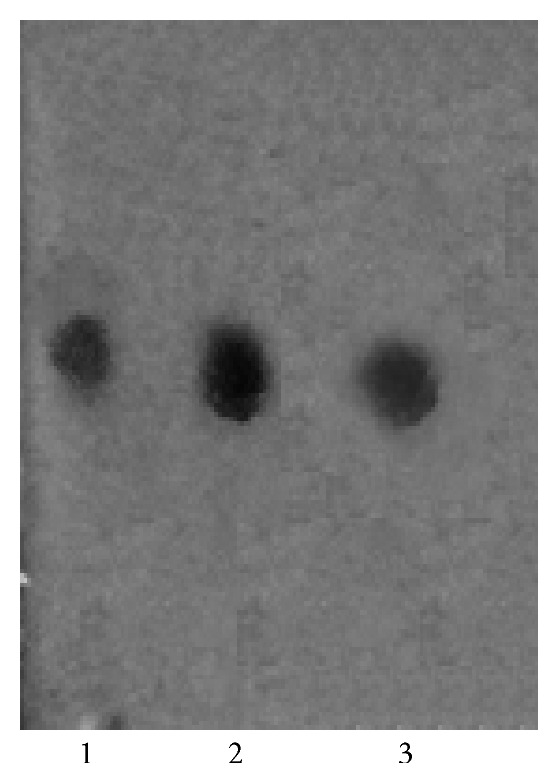
The thin layer chromatography spectrums of sample hydrolysate. Note: Lane 1, standard sample of L-glutamic acid; Lanes 2 and 3, hydrolyzed sample of *γ*-PGA.

**Figure 6:**
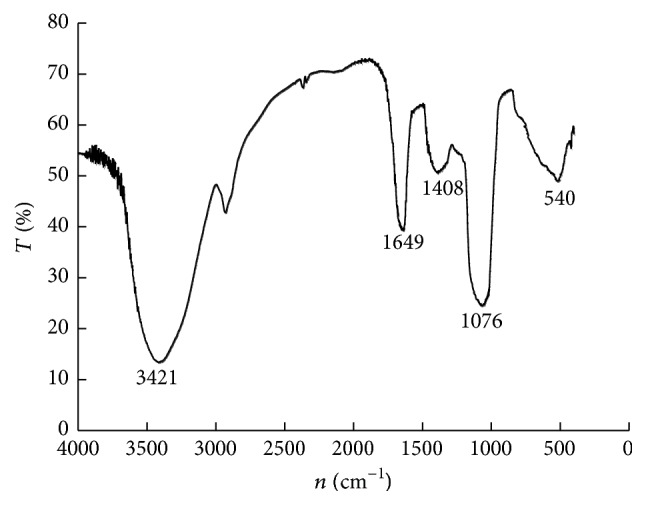
Analysis of FT-IR spectrum of the *γ*-PGA sample. The absorption peak at 3,421 cm^−1^ was the symmetric stretching vibration band of N-H; 1,649 cm^−1^ was the asymmetric stretching vibration band of –CONHR; 1,408 cm^−1^ was the symmetric stretching vibration band of COOH; 1,076 cm^−1^ was the hallmark peak representing the presence of aliphatic hydrocarbons, -CH_2_ or -CH_3_ (flexural vibration); 1,000 cm^−1^–500 cm^−1^ were caused by (CH_2_)_*n*_ (*n* > 4) planar rocking vibration, as well as in-plane bending vibration.

**Figure 7:**
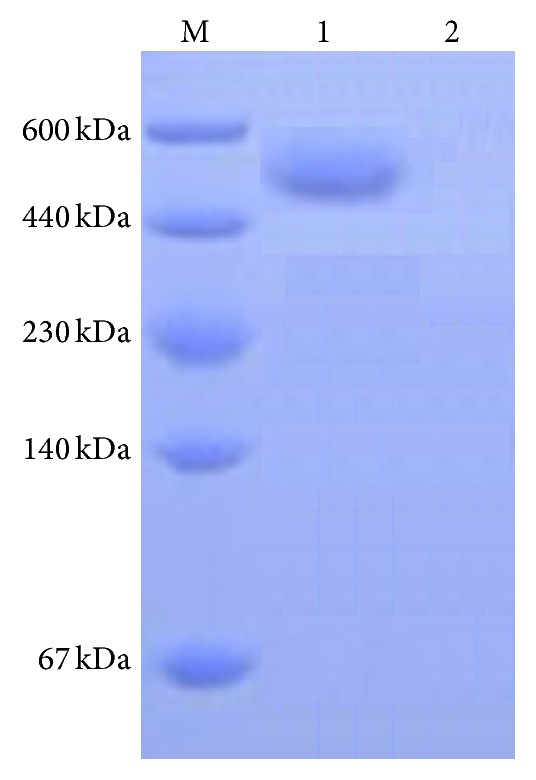
SDS-PAGE analysis of product of pWB980-*pgs*BCA. Note: Lane M, marker, high molecular weight standard protein (TakaRa); Lane 1, *γ*-PGA samples obtained and purified from the fermentation broth; Lane 2, control,* Bacillus subtilis* WB600.

Figure 5 shows the TLC results of the hydrolysate samples observed under ultraviolet light, wherein, after the acid hydrolysis of *γ*-PGA, no other band was observed on the silica gel plates but only single spots of uniform color intensity. Its retention (*R*_*f*_) value was consistent with that of the standard, glutamate spots, indicating that the hydrolysates had no other amino acids and other protein impurities. These hydrolysates were in single form, solely consisting of pure glutamic acid.  Figure 6 shows the infrared (IR) spectroscopy of *γ*-PGA. The absorption peak at 3,421 cm^−1^ was the symmetric stretching vibration band of N-H; and the absorption peak at 1,649 cm^−1^ was the asymmetric stretching vibration band of an amide group, -CONHR. Both peaks were the main indicators used in the identification of amides and for the presence of amide groups in *γ*-PGA molecules. The absorption peak at 1,408 cm^−1^ was the symmetric stretching vibration band of COOH; the absorption peak at 1,076 cm^−1^ was the hallmark peak representing the presence of aliphatic hydrocarbons, -CH_2_ or -CH_3_ (flexural vibration), in the molecular structure; and the absorption peaks within the range of 1,000 cm^−1^–500 cm^−1^ were caused by the (CH_2_)_*n*_ (*n* > 4) planar rocking vibration, as well as in-plane bending vibration. The spectral characteristics of recombinant *γ*-PGA in fermentation broth was consistent with those of the standard *γ*-PGA's IR spectroscopy, indicating that the sample obtained in the present study contained the N-H and C=O functional groups, as well as the aliphatic hydrocarbon structure (CH_2_)_4_ of the *γ*-PGA [17], thereby confirming that the sample was *γ*-PGA. The molecular weight of the *γ*-PGA sample obtained after the fermentation, isolation, and separation of recombinant strain* Bacillus* WB600-*pgs*BCA was determined using SDS-PAGE.  Figure 7 shows that the molecular weight of the *γ*-PGA was between 500 and 600 kDa and occurred as aggregates of a multimolecular mass, but not of a single molecular composition.

## 4. Discussion and Conclusions

The present study evaluated the cloning and expression of *γ*-PGA synthase gene* pgs*BCA in* B. subtilis* and used plasmid pWB980 to construct the recombinant expression vector, pWB980-*pgs*BCA, and to further transfer the recombinant expression vector into* B. subtilis* WB600. The P43 promoter of pWB980 induced the expression of* pgs*BCA; then the host cells of this expression vector showed a capacity to synthesize *γ*-PGA, and the product yield of *γ*-PGA reached ≥1.74 g/L. The isolated and purified *γ*-PGA sample from the fermentation broth was confirmed to have a single form of hydrolysates that solely consisted of pure glutamic acid. This result matched the characteristics of the standard *γ*-PGA's IR spectroscopy and showed the aggregates of a multimolecular mass, with a molecular weight ranging between 500 and 600 kDa.

The present study used* B. subtilis* as the expression host, and the* pgs*BCA gene originated and was expressed in* B. subtilis*. The *γ*-PGA synthase system is better positioned in the cell membrane (as shown in Section 1). Therefore, the synthetic yield and molecular weight of *γ*-PGA produced in* B. subtilis* were as high as ≥1.74 g/L and between 500 and 600 kDa, two features that are consistent with or even higher than the expression system of* E. coli* and* B. subtili*s that had previously been described to have high expression efficiency [18–20]. The molecular weight of *γ*-PGA especially expressed in this host is the highest in the existing report [21–24]. The recombinant expression vector, pWB980-*pgs*BCA, in the present study contained the P43 promoter. Therefore, the costly use of isopropyl *β*-D-1-thiogalactopyranoside (IPTG), D-xylose, and L-arabinose as an inducer to secrete the* pgs*BCA into the extracellular fermentation broth is circumvented using the methodology developed in the present study. This technique may also be potentially used in industrial production as it can increase the stability of products, simplify the purification work, and have more obvious application potential advantage.

Although the constructed recombinant bacteria* Bacillus* WB600-*pgs*BCA showed the capacity to synthesize *γ*-PGA, our results still could not match the highest synthetic yield of *γ*-PGA (40–50 g/L) that is induced by the fermentation of mutated bacteria [25, 26]. Therefore, our next research study will focus on introducing hemoglobin, other exogenous genes, or certain control sequences to efficiently synthesize and express *γ*-PGA and to increase the bacterial concentration, oxygen uptake, or endogenous synthase expression, thereby ultimately increasing *γ*-PGA yield [27, 28]. Alternatively, we will knock out genes of degrading enzymes in *γ*-PGA-producing strains to reduce *γ*-PGA degradation, thereby increasing *γ*-PGA yield [29]. Therefore, our future research direction and goal will focus on establishing and modifying our current engineered strains through genetic engineering to improve its performance and further increase *γ*-PGA yield, thereby laying the foundation for the industrial production of high-yielding *γ*-PGA engineered bacteria based on the* B. subtilis* expression system.
